# Distribution of bovine cysticercosis prevalence in the southeastern districts of Botswana from 2015 to 2016

**DOI:** 10.14202/vetworld.2022.368-373

**Published:** 2022-02-17

**Authors:** Batatu Mazhani, Elly Masitha, Mpho Ntwaetsile, Ketshephaone Thutwa, Kerapetse Sehularo

**Affiliations:** 1 Department of Veterinary Sciences, Faculty of Animal and Veterinary Sciences, Botswana University of Agriculture and Natural Resources, Private Bag 0027, Gaborone, Botswana; 2 Meat Industry Training Institute, Faculty of Animal and Veterinary Science, Botswana University of Agriculture and Natural Resources, Private Bag 0027, Gaborone, Botswana; 3 Department of Animal Sciences, Faculty of Animal and Veterinary Science, Botswana University of Agriculture and Natural Resources, Private Bag 0027, Gaborone, Botswana

**Keywords:** bovine cysticercosis, prevalence, geospatial distribution, meat industry, seasonal occurrence

## Abstract

**Background and Aim:**

Bovine cysticercosis is defined as a foodborne parasitic zoonotic disease of cattle caused by the larval stage of the human tapeworm *Taenia saginata*. In Botswana, bovine cysticercosis has inflicted major economic consequences on the beef industry due to downgrading, condemnation, or treatment of infected carcasses. Thus, in this study, we aim to (1) estimate the prevalence of bovine cysticercosis in Botswana’s southeastern districts, (2) describe the distribution of bovine cysticercosis through geospatial mapping, and (3) investigate the effect of seasonality on bovine cysticercosis occurrence.

**Materials and Methods:**

A retrospective study was conducted using abattoir records of cattle slaughtered from August 2015 to July 2016. In total, 13 licensed non-export abattoirs were selected for this study, wherein 26,827 cattle were slaughtered during this period. Detection of cysticerci from the carcass and offal was carried out by meat inspectors visually during meat inspection. Prevalence of bovine cysticercosis was calculated for the extension areas and veterinary districts for each month and form there used to establish its distribution and seasonality. Data were analyzed using SPSS version 25.0.

**Results:**

The prevalence of bovine cysticercosis in the southeastern districts of Botswana during this study period was determined to be 6.2%. The prevalence in the veterinary districts differed significantly at p<=0.000. Seasonality did not have a significant (p=0.651) effect on the prevalence of bovine cysticercosis. Geospatially, areas with greater than 8% prevalence were mainly located in the southernmost part of the study area.

**Conclusion:**

The prevalence of bovine cysticercosis was 6.2% during the study period. No previous studies on cysticerci prevalence in the study area was conducted; thus, it was not possible to determine whether there has been an increase or decrease in terms of prevalence rate. Therefore, the results of this study can be used as a baseline for the prevalence of cysticerci in the study area.

## Introduction

Bovine cysticercosis is described as a foodborne parasitic zoonotic disease of cattle caused by the larval stage of the human tapeworm *Taenia saginata*. The life cycle of the parasite occurs in both humans and cattle. The larvae are meat borne, and the adult stage develops only in the intestine of the human host (obligate). When expelled into the surrounding environment, infective eggs from humans can contaminate feed and water. On ingestion of infective eggs by a bovine (intermediate host), an embryo (or oncosphere) hatches from the egg and penetrates the host’s intestinal mucosa to enter its circulatory system [1]. Once in the muscle or tissue, the embryo then develops into a cysticercus and becomes infective to a human host after about 10-12 weeks [1]. Detection of cysticerci in cattle is conducted through routine meat inspection. However, studies have shown that the prevalence estimates using this detection method underestimate parasite prevalence by a factor of at least 3-10 [2]. Cysticerci are found predominantly in cardiac and skeletal musculature, but occasionally in other sites, including liver, lungs, kidneys, and lymph nodes [1]. Surveillance of bovine cysticercosis through meat inspection is carried out in all licensed abattoirs in Botswana [3].

The detention or condemnation of meat due to cysticerci could often result in economic losses to all beef industry players. The Botswana Meat Commission reported a loss of Botswana Pula (BWP) 83,648,504 in 2013 due to refrigeration costs and decreased market value of infected carcasses [4]. Despite the considerable significance of this disease to the economy, the study studies on the epidemiology of bovine cysticercosis in Botswana have remained scarce [5,6]. In fact, in all of Southern Africa, studies examining the prevalence of this disease have remained to be limited [7-9].

Considering the major contribution of the beef subsector to Botswana’s agricultural share of the country’s gross domestic product [10], this study aimed to (1) determine the prevalence of bovine cysticercosis in Botswana’s southeastern districts by examining records of cattle slaughtered from August 2015 to July 2016 in licensed non-export abattoirs, (2) describe the distribution of bovine cysticercosis through geospatial mapping, and (3) investigate the effect of seasonality on the occurrence of bovine cysticercosis. The results of this study can be used by government authorities and industry leaders as a guide for the implementation of strategies to prevent and control the spread of bovine cysticercosis.

## Materials and Methods

### Ethical approval

Ethical approval for this study was not required. Data used in this study were obtained from slaughter facilities licensed under the regulatory authority of the Department of Veterinary Services under the Ministry of Agriculture and Food Security. All slaughtering complied with animal welfare ethical requirements as stated in the standards, regulations, and laws of the Government of Botswana. Approval for the research was thus granted by the Director of Veterinary Services (Reference V 6/1/31 IV [19]).

### Study period, location, and sampling

This study covered cattle slaughtered at licensed non-export abattoirs in the southeastern districts of Botswana from August 2015 to July 2016. These administrative districts are South, South East, Kweneng, and Kgatleng, covering nine veterinary districts Goodhope, Kanye, Jwaneng, Lobatse, Gaborone, Ramotswa, Mochudi, Molepolele, and Letlhakeng. Respective veterinary districts are further divided into extension areas. Human population for the respective veterinary districts in the study were Kanye (47,007), Gaborone (231,592), Jwaneng (18,008), Molepolole (66,466), Lobatse (29,007), Goodhope (6362), Mochudi (44,815), Letlhakeng (7229), and Ramotswa (28,952) [11].

A total number of 13 licensed non-export abattoirs were selected for the study. These abattoirs make up 76% of abattoirs operational during the study period. The catchment for the abattoirs is predominantly communal areas. Abattoirs excluded were those that slaughtered less that 5 cattle per week, those not operational during the study period, and those with unreliable and incomplete records.

Detection of cysticerci was performed according to the regulations set out by the Department of Veterinary Services (Republic of Botswana Laws, 2007). Information recorded in the abattoir records, although not standardized, included date of slaughter, district of origin, extension area of origin, total number of cattle slaughtered, total number of carcasses detected with cysticerci, total number of carcasses treated, and total number of carcasses condemned. The place of origin in terms of the veterinary district and extension area was of interest to our study. Cattle originating from veterinary districts outside of the study area were excluded in the analysis. The Botswana Animal Information and Traceability System was used in some instances to obtain missing information.

### Data collection

Existing monthly abattoir records of cattle slaughtered were collected and analyzed to determine bovine cysticercosis prevalence; as per the data collected, 26,827 slaughtered cattle were recorded.

### Statistical analysis

The prevalence was defined as the number of cattle identified as harboring cysticerci during meat inspection divided by the number of slaughtered cattle [12]. This was expressed as a percentage for each extension area and veterinary districts within the area of study. One-way analysis of variance in Statistical Package for the Social Sciences V 25.0 (IBM Corp., NY, USA) was used to determine whether bovine cysticercosis prevalence differed between veterinary districts of Botswana. As the data were not normally distributed, it was log_10_ transformed to approximate normality. Since there were data entries with values 1 was added to all the prevalence values before log_10_ transformations. However, the averages presented in the results section are untransformed. The Games-Howell *post-hoc* test was used to compare the means.

## Results

The overall prevalence of bovine cysticercosis in the study area was 6.2%. The prevalence for the respective administrative districts was observed to be 7.3% (South), 4.5% (Kgatleng), 5.5% (Kweneng), and 6.0% (Southeast).

### Prevalence rate in the veterinary districts

**Figure-1:**
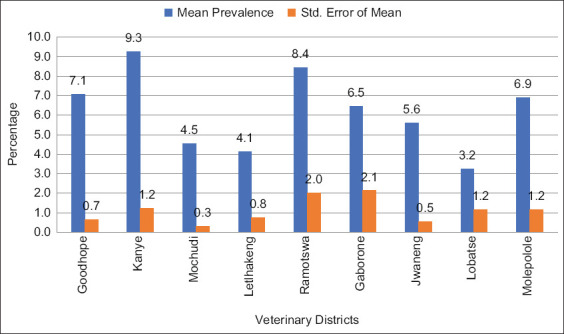
The mean prevalence of bovine cysticercosis in the veterinary districts (n=9) with standard errors from 2015 to 2016.

The prevalence of bovine cysticercosis was noted to differ significantly (p=0.000) between the studied veterinary districts (Figure-1). As per our findings, Lobatse had the lowest prevalence (3.2%), while Kanye had the highest prevalence (9.3%) in the areas examined. According to our Games-Howell*post ho*c analysis, Goodhope had a significantly higher bovine cysticercosis prevalence (p=0.000) than Letlhakeng. The prevalence rate of this disease was also significantly higher in Kanye than in Letlhakeng (P=0.003) and Mochudi (P=0.000),

### Prevalence in extension areas

**Figure-2:**
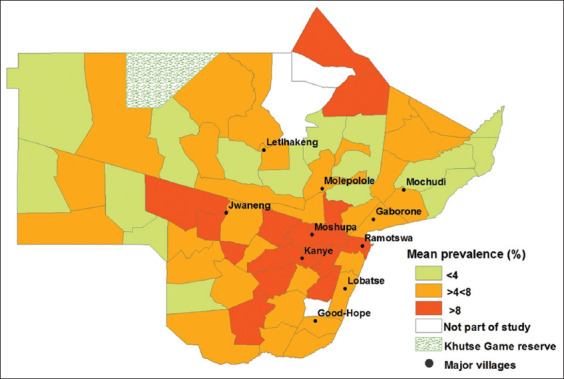
Geospatial distribution of the mean prevalence of bovine cysticercosis in extension areas (n=78) in the southeastern districts of Botswana from 2015 to 2016 [Source: Department of Veterinary Services, IT Department. Graphics program: ArcGIS 10.4].

The prevalence of bovine cysticercosis in extension areas was deemed significant (p=0.000). The prevalence was highest in Mmankgodi at 19% and lowest in Kokong and Morwamusu extension area at 0% (Table-1). Geospatial distribution demonstrated that most extension areas with high prevalence were concentrated in the Kanye district (Figure-2). In addition, the prevalence of bovine cysticercosis has progressively decreased east and west of the Kanye district (Figure-2).

**Table 1:** Mean prevalence of bovine cysticercosis in extension areas (n=78) in the southeastern districts of Botswana.

Extension area	Mean (%) ±S.E	Extension area	Mean (%) ±S.E	Extension area	Mean (%)±S.E
Mmathethe	7.888±0.572	Artesia	5.282±1.202	Thankane	3.207±1.722
Metlobo	8.790±2.118	Dikgonnye	4.8000±1.076	Sekoma	2.644±1.048
Pitsane Molopo	6.032±1.380	Oodi	5.006±1.235	Maokane	5.471±1.236
Mokatako	10.604±2.951	Leshibitse	6.072±1.222	Mokhomma	8.238±1.901
Pitsane	3.897±2.036	Ramotlabaki	3.775±1.252	Jwaneng	9.578±3.443
Digawana	10.810±3.440	Olifantsdrift	3.132±0.897	Samane	6.662±1.557
Ramatlabama	2.543±1.362	Maboane	5.562±1.623	Sese	7.737±1.481
Pelotshetlha	7.727±2.971	Moshaweng	6.315±4.177	Tsonyane	8.281±2.234
Goodhope	5.452±1.506	Ngware	5.786±3.952	Keng	2.850±1.027
Hebron	7.912±1.936	Ditshegwane	0.571±0.571	Khakhea	4.732±2.168
Mabule	6.210±1.231	Salajwe	4.284±3.822	Kokong	0.000±0.000
Moshupa	15.010±3.627	Kudumelapye	5.251±2.374	Morwamusu	0.000±0.000
Gasita	4.363±1.542	Letlhakeng	7.323±3.227	Lobatse	3.250±1.170
Kanye	13.213±3.315	Dutlwe	4.227±2.281	Gabane	4.124±1.116
Segwagwa	10.746±2.546	Monwane	1.122±1.122	Mogoditshane	4.436±2.152
Molapowabojang	9.908±4.254	Sorilatholo	10.000±5.774	Lephepe	14.679±5.892
Ranaka	11.950±8.111	Takatokwane	3.472±2.261	Medie	5.425±1.782
Sesung	4.901±2.885	Botlhapatlou	0.760±0.760	Boatlaname	12.754±8.899
Lorolwane	2.911±1.570	Motokwe	1.335±0.901	Lentsweletau	2.255±1.827
Pitseng	11.267±2.408	Mogobane	10.058±4.268	Hatsalatladi	12.307±6.613
Selokolela	9.857±4.075	Otse	3.175±1.762	Kopong	3.333±3.333
Kgomodiatshaba	7.436±0.772	Ramotswa	11.916±3.427	Thamaga	3.175±2.100
Bokaa	3.955±0.806	Gaborone	10.490±3.569	Mmankgodi	19.033±4.807
Mochudi	4.757±0.779	Tlokweng	2.774±2.047	Sojwe	3.634±1.987
Malolwane	3.260±0.797	Mahotshwane	5.480±1.379	Kubung	9.109±2.627
Malotwane	2.537±0.550	Mabutsane	7.414±2.317	Molepolole	4.029±3.183

### Prevalence of bovine cysticercosis in different months

**Figure-3:**
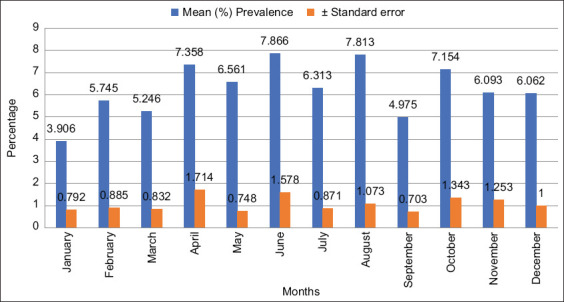
Monthly mean percentage prevalence of bovine cysticercosis in the southeastern districts of Botswana from 2015 to 2016.

Overall, the month wherein a record was taken did not significantly affect (p=0.651) bovine cysticercosis prevalence in the study area. However, January had the lowest prevalence (3.9%), whereas June had the highest prevalence rate (7.9%) (Figure-3). Parasite prevalence during the wet season, from October to March, tended to be lower (5.7%) than that of the dry season (6.8%), from April to September.

## Discussion

This study indicates that the overall mean prevalence of bovine cysticercosis in the southeastern districts of Botswana was 6.2% during the study period. No previously published bovine cysticercosis prevalence studies have been conducted in this study area, to which we can compare the results of our study. However, a bovine cysticercosis incidence survey was previously performed using 10-year data from across Botswana collected by the export abattoir Botswana Meat Commission [5]. In that study, bovine cysticercosis incidence ranged from 12% to 15%.

The bovine cysticercosis prevalence in our study was higher than that of other African countries. A prevalence of 0.7% was reported in South Africa [8], 1.6% in Zimbabwe [9] and Egypt [13], and 2.6% in Ethiopia [14]. Conversely, this study’s prevalence rate was lower than that in a study conducted in East Ethiopia where disease prevalence was at 19.7% [15].

The prevalence of bovine cysticercosis in this present study was also higher than that of countries outside of Africa, namely, Iran (1.71%) [12], Brazil (0.41%) [16], France (0.142%) [17], and Denmark (0.06%) [18].

Bovine cysticercosis prevalence in the Kgatleng administrative district was lower than in the other administrative districts. This is possibly due to farmers’ awareness of *Taenia saginata* control measures and a generally positive attitude toward prevention and control of this disease in the district [6].

This study has also revealed that the prevalence rates of bovine cysticercosis in Kanye, Goodhope, and Ramotswa veterinary districts are higher than that of Gaborone, which is in contrast to the results from Rossi *et al*. [19], who found that densely human populated areas had higher bovine cysticercosis prevalence. One possible explanation for this conflicting result is that Gaborone, as a city, has good sewage and sanitation systems and thus has a minimized risk of environmental contamination; also, Gaborone has low cattle populations. On the other hand, Kanye, Goodhope, and Ramotswa, as villages, have poorly developed sewage systems and high cattle populations in close proximity to humans, which could be possible risk factors for the high occurrence rate of bovine cysticercosis [20]. Furthermore, the Kanye veterinary district is often used for leisure activities that occur in cattle pastures where sanitation systems are poor, posing a higher risk for contracting bovine cysticercosis [20].

Although not statistically significant, prevalence during the wet seasons (November-March) was slightly lower than that during the dry seasons. These findings were consistent with other studies carried out in countries with similar climatic conditions as those in the study area [8,9]. The slight differences in prevalence may be due to the effect of temperature and humidity on the survivability of *T. saginata* eggs [21]. Damp and temperate climates provide hospitable conditions for *T. saginata* eggs for roughly 10 months [1]. Climatic conditions in Botswana during the wet season are characterized by high temperatures that potentially impact the survivability of *T. saginata* eggs. In contrast, an increase in bovine cysticercosis incidence during drought season has been attributed to high cattle-human contact caused by competition for water between humans and livestock [5].

## Conclusion

Bovine cysticercosis prevalence in Botswana’s southeastern districts was high (6.2%) relative to Zimbabwe and South Africa and thus warrants immediate action. Since there is no record of previous prevalence studies from Botswana, the results of this study can be used as a baseline for futureresearch in the study area. Furthermore, our results will be valuable for informing governmentfor the policies and providing information on where to target intervention measures in the control and prevention of bovine cysticercosis. As illustrated geospatially, priority areas should be those with a prevalence rate greater than 8%. Finally, future epidemiological studies to determine the local risk factors within different veterinary districts should be considered, as this was not evaluated in the present study.

## Authors’ Contributions

BM, EM, and KS: Conceived and designed the study. MN: Collection of data. BM and EM: Interpreted results and revised map. KT: Analyzed the data and revised the manuscript. BM: Drafted and revised the manuscript. All authors have read and approved the final manuscript.
